# Notfall-Apps: vergeudete Zeit oder sinnvolles Instrument in der präklinischen Versorgung von Traumapatienten?

**DOI:** 10.1007/s00063-020-00675-2

**Published:** 2020-04-08

**Authors:** C. Kaczmarek, H. Andruszkow, C. Herren, M. Pishnamaz, F. Hildebrand, A. Röhl, P. Lichte

**Affiliations:** 1 Klinik für Unfall- und Wiederherstellungschirurgie, Uniklinik RWTH Aachen, Pauwelsstraße 30, 52074 Aachen, Deutschland; 2 St. Elisabeth-Krankenhaus Geilenkirchen, Geilenkirchen, Deutschland; 3 Klinik für Anästhesiologie, Uniklinik RWTH Aachen, Aachen, Deutschland

**Keywords:** Polytrauma, ICE-Nummer, Trauma, Smartphone, Notfall-ID, ICE contact, Medical alert ID, Patient identification system, Trauma, Smartphone

## Abstract

**Hintergrund:**

Um in Notfallsituationen eine effektive Versorgung des Patienten zu gewährleisten, sind Informationen über Vorerkrankungen und bisherige Medikamenteneinnahme essenziell. Daher haben Smartphonehersteller entsprechende Anwendungssoftware (App) entwickelt, auf die im Notfall zugegriffen werden kann, um jene Informationen zu erhalten. Ziel der Studie war es herauszufinden, ob Notfall-Apps von Smartphoneinhabern aktiv genutzt werden und ob diese in Notfallsituationen von den behandelnden Notärzten eingesehen werden.

**Methode:**

Zur Datenerhebung wurde eine anonymisierte Umfrage über das Nutzungsverhalten der Notfall-Apps bei verunfallten Patienten der unfallchirurgischen Ambulanz eines universitären Maximalversorgers über einen Zeitraum von 3 Monaten durchgeführt. Parallel fand eine Befragung von Notärzten verschiedener Standorte zu ihren beruflichen Erfahrungen mit den Apps statt.

**Ergebnisse:**

Insgesamt wurden 192 Patienten und 103 Notärzte befragt. Die Notfall-Apps waren 45 % (*n* = 79) der Befragten nicht bekannt; nur bei 10 % (*n* = 19) der Befragten war die App mit Daten hinterlegt. Weiterhin zeigte sich, dass insgesamt 21 % (*n* = 41) der Personen einen Notizzettel mit Vorerkrankungen und Medikamenten bei sich trugen. Von den Befragten Ärzten gaben 42 % (*n* = 44) an, schon einmal von der App gehört zu haben; nur 6 % (*n* = 5) durchsuchten jedoch routinemäßig bei nichtansprechbaren Patienten das Smartphone. Erfolgreich genutzt wurde die App bisher nur von 14 % der Ärzte (*n* = 14).

**Schlussfolgerung:**

Aufgrund der geringen Bekanntheit erscheint es in zeitkritischen Situationen nicht empfehlenswert, das Smartphone der Patienten nach Notfall-Apps zu durchsuchen. Bei Patienten über 55 ist es zurzeit erfolgsversprechender, die Brieftasche nach Informationen zu Vorerkrankungen zu kontrollieren.

## Abstract (en)

**Background:**

In order to provide safe care to a patient in an emergency situation, it is useful to know something about the patient’s previous medical conditions and medication. For this very reason smartphones have been equipped with so-called emergency apps (e.g. medical-ID, emergency-ID). The aim of our study was to find out whether the owners of smartphones are using the apps and whether medical professionals are trying to access this information.

**Methods:**

We conducted a survey among patients in our outpatient clinic at a level one trauma center. We collected data over 3 months regarding the usage behavior of the aforementioned apps. We simultaneously asked emergency physicians at various hospitals about their experiences with these apps.

**Results:**

We were able to interview 192 patients and 103 emergency physicians. The emergency apps were unknown to 45% (*n* = 79) of the respondents; only 10% (*n* = 19) of the respondents had the app with data stored. Furthermore, it was found that a total of 21% (*n* = 41) of the persons carried a note on themselves with previous illnesses and medication. Of the surveyed physicians, 42% (*n* = 44) stated that they had heard of the app before; however, only 6% (*n* = 5) routinely searched the smartphone for relevant information in the case of nonresponsive patients. Only 14% of physicians (*n* = 14) have successfully used the app so far.

**Conclusion:**

The collected data show that the emergency apps are still unknown to many patients and emergency physicians alike. Due to the low distribution it does not seem to be recommendable to search the smartphone for the apps in time-critical situations after accidents. For patients over 55 years of age, it currently seems more promising to search their wallets for information regarding their previous illnesses.

## Einleitung

Nach dem Terroranschlag auf mehrere Busse in London im Jahr 2005 initiierte ein britischer Rettungssanitäter eine Kampagne, die Menschen zur Hinterlegung eines „In-case-of-emergency“-Kontakts, der sog. ICE, in ihrem Mobiltelefon (Handy und Smartphone) anregen sollte [1]. Unter dem Kürzel ICE sollte die Telefonnummer eines Angehörigen eingespeichert werden, der im Notfall als Kontaktperson angerufen werden konnte [2]. Angehörige könnten auf diese Weise zügig benachrichtigt werden und womöglich wichtige Informationen über die zu behandelnde Person beitragen. Außerdem sollte eine solche Nummer die Identifikation von Verletzten bei einem Massenanfall von Verletzten (MANV) erleichtern.

**Figure 1:**
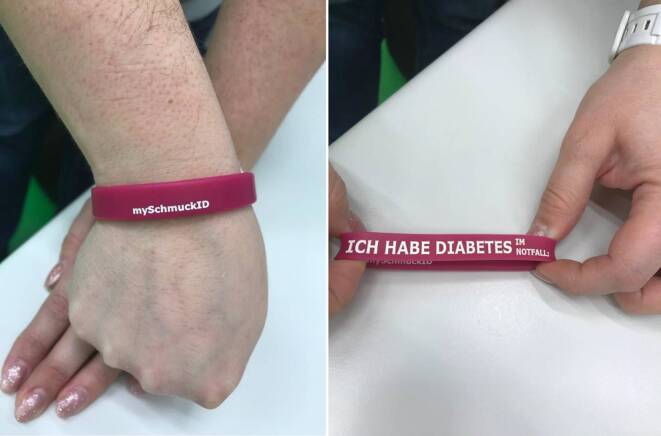

Zeitgleich zur ICE-Kampagne entwickelten sich weitere Aktionen mit einem ähnlichen Ansatz. Dabei wurden beschriftete Armbänder beworben, die es Rettungshelfern ermöglichen sollten, Notfallsituationen schneller korrekt einordnen zu können. Auf diesen Armbändern war z. B. vermerkt, ob ein Patient an Diabetes oder Epilepsie leidet. Für diese Maßnahme existieren jedoch weiterhin keine einheitlichen Regelungen, sodass jede Fachgesellschaft eigene Erkennungsfarben und -designs verwendet. Für den Helfer ist es somit nicht auf Anhieb möglich, die Armbänder zu identifizieren und zu interpretieren ([3, 4]; Abb. 1). Zudem tragen insbesondere ältere Patienten, die häufiger Vorerkrankungen aufweisen und dementsprechend regelmäßig Medikamente einnehmen, oft einen Medikationsplan oder einen Zettel mit Vorerkrankungen bei sich [5]. Patienten, die mehr als 3 Medikamente über einen Zeitraum von mehr als 28 Tagen verschrieben bekommen, erhalten seit dem 01.10.2017 von ihrem Hausarzt außerdem einen Bundesmedikationsplan. Auch dieser wird häufig in der Brieftasche mitgeführt [6].

Die allgemeine Nutzung von Sperrbildschirmen auf Smartphones führt mittlerweile dazu, dass die hinterlegten ICE-Nummern nicht mehr zugänglich sind. Sogenannte Notfall-Applikationssoftware (App) und Notfallpässe speziell für die Smartphones wirken dieser Problematik entgegen, da hierdurch die für den Notfall im Smartphone hinterlegten Daten ohne Kenntnis der persönlichen Identifikationsnummer (PIN) einzusehen sind. Je nach Hersteller werden diese Apps derzeit unter unterschiedlichen Bezeichnungen (Notfallpass, Notfall-App, Notfallinformationen, Notfall etc.) geführt und vermarktet. Alle Hersteller ermöglichen dem Nutzer die Speicherung und Bereitstellung unterschiedlicher Daten wie Größe, Gewicht, Vorerkrankungen, Blutgruppe, Medikamente und Notfallkontakte. Ein einheitlicher Standard für die Gestaltung und Bedienung dieser Apps besteht bisher nicht.

Zurzeit liegen keine uns bekannten Studien vor, die sich mit dem Notfallpass oder vergleichbaren Apps auseinandergesetzt haben [3]. Es liegen keine Kenntnisse darüber vor, wie viele Menschen entsprechende Apps nutzen, ob die dort hinterlegten Daten valide sind und inwiefern diese von Notärzten genutzt werden. Dementsprechend liegt dieser Studie vor allem die Analyse und Klärung folgender Fragestellungen zugrunde:

1. Wie viele Smartphonebesitzer haben eine Notfall-App eingerichtet?
2. Werden Notfall-Apps von Notärzten genutzt bzw. wird nach den dort enthaltenen Informationen gezielt gesucht?

## Methode

Die vorliegende Studie wurde durch die Ethikkommission der medizinischen Fakultät der Uniklinik Aachen genehmigt (EK 045/18). Über einen Zeitraum von 3 Monaten wurde eine Befragung von Patienten der unfallchirurgischen Klinik eines Universitätsklinikums und Level-1-Traumazentrums zu dem Nutzungsverhalten ihrer Smartphones in Bezug auf Notfall-Apps durchgeführt. Nach Einwilligung der Patienten zur Studienteilnahme erfolgte die Umfrage anonymisiert mittels Fragebogen. Die Befragten befanden sich aufgrund von Unfallverletzungen in der laufenden ambulanten Behandlung und wurden zufällig ausgewählt. Zusätzlich zum Nutzungsverhalten des Smartphones wurde nach der Meinung zur Speicherung von Notfalldaten auf der Krankenkassenkarte gefragt. Der vollständige Fragenkatalog ist in Tab. 1 dargestellt.

**Table 1:** 

Wie alt sind Sie?
Geschlecht: M/W
Besitzen Sie ein Smartphone? Wenn ja, welches?
Ist Ihr Notfallpass (iPhone) aktiv bzw. nutzen Sie eine Notfall-App (Android)?
Würden Sie – jetzt wo Sie wissen, dass es sie gibt – diese App benutzen?
Haben Sie in Ihrer Brieftasche eine Medikamentenliste oder Informationen über Ihre Erkrankungen?
Haben Sie in Ihrem Telefon einen Kontakt hinterlegt, den man in Notfall anrufen soll, auch ICE-Nummer genannt?
Haben Sie einen PIN in Ihrem Telefon?
Sollten medizinische Daten auf der Krankenkassenkarte gespeichert werden dürfen?

Um zu evaluieren, ob ein lebensbedrohliches Unfallereignis das Nutzungsverhalten bezüglich der Notfall-Apps oder ICE-Nummer beeinflusst, wurden gezielt 30 ehemals polytraumatisierte Patienten (ISS [Injury Severity Score] > 15) zusätzlich in die Studie eingeschlossen.

Zudem wurden Ärzte, die regelmäßig als Notarzt tätig sind, zu ihren Erfahrungen mit den Notfall-Apps befragt. Die teilnehmenden Ärzte wurden entweder anonym mittels Fragebogen oder über die Onlineplattform *surveymonkey.com *[7] befragt (Tab. 2).

**Table 2:** 

Sie sind: Assistenzarzt, Facharzt, Oberarzt, Chefarzt?
Haben Sie schon mal von dem Notfallpass* (Apple iPhone) bzw. NotfallApps* (Android) gehört?
Schauen Sie routinemäßig bei nichtansprechbaren Patienten (Schockraum, Notarzt, Intensivstation) im Smartphone nach Informationen?
Haben Sie schon einmal eine solche App erfolgreich genutzt?
Schauen Sie routinemäßig bei nichtansprechbaren Patienten (Schockraum, Notarzt, Intensivstation) in der Brieftasche oder im Handy nach Informationen?

Die Studienergebnisse wurden mithilfe des statistischen Programms SPSS (IBM Corp. Released 2017; IBM SPSS Statistics for Windows, Version 25.0.Armonk, NY: IBM Corp.) analysiert. Neben der deskriptiven Statistik wurden Unterschiede mittels Χ^2^-Tests untersucht. Korrelationen wurden mittels Spearman-Rho-Test analysiert. Die statistische Signifikanz wurde bei einem *p* < 0,05 definiert.

## Ergebnisse

Von den 195 befragten Patienten konnten insgesamt 192 Fragebögen ausgewertet werden (98,5 %), 3 Fragebögen waren nur lückenhaft oder fehlerhaft ausgefüllt. Es nahmen insgesamt 106 Männer und 86 Frauen teil. Das Durchschnittsalter betrug 45 (±15) Jahre. Von den Befragten nutzten 174 ein Smartphone, 73 davon ein iPhone und 101 ein Smartphone mit einem Android-Betriebssystem. Die Notfall-App war bei 10 % der Befragten (*n* = 19) mit Daten hinterlegt. Von den 192 Befragten gaben 79 Personen (41 %) an, noch nie von der Notfall-App gehört zu haben. Die Frage, ob die Befragten nun, da sie von der App gehört haben, diese benutzen würden, wurde von 60 % bejaht. Vom Gesamtkollektiv gaben 21 % (*n* = 41) Personen an, eine Liste mit Vorerkrankungen und Medikamenten bei sich zu tragen. Das Durchschnittsalter betrug hier 56 (±11) Jahre und lag damit signifikant höher als das des Gesamtkollektivs. Von älteren Patienten (>55 Jahre) wurden diese Listen signifikant häufiger genutzt als von jüngeren (70 % vs. 11 %; *p* < 0,001).

Eine im Smartphone hinterlegte Notfallnummer hatten – mit einem Durchschnittsalter von 47 (±14) Jahren − 27 % (*n* = 52) gespeichert. Jedoch gaben 86 % (*n* = 45) dieser Patienten an, ihr Smartphone mit einer PIN gesichert zu haben. Im Gesamtkollektiv hatten ebenfalls 84 % aller Befragten (*n* = 161) ihr Smartphone durch eine PIN geschützt. Eine statistisch relevante Korrelation zwischen Alter und der Nutzung der Notfall-App wie auch der ICE-Nummer gab es nicht.

Das Speichern von Daten auf der Krankenkassenkarte befürworteten 52 % (*n* = 100) der Befragten, 43 % (*n* = 83) waren dagegen und 5 % (*n* = 9) gaben an, dass es ihnen egal sei.

Von den 30 befragten Patienten, die in der Vergangenheit ein Polytrauma erlitten hatten, gaben alle an, bei ihrem Unfall ein Smartphone mitgeführt zu haben. Ihre Einstellung zu den Notfall-Apps nach einem Unfall haben 36% (*n* = 11) der Befragten geändert. Nur 6 % (*n* = 2) gaben an, eine Notfall-App nachträglich aktiviert zu haben. Insgesamt hatten in dieser Gruppe 13 % (*n* = 4) eine Notfall-App aktiviert, 2 der Befragten hatten diese bereits zum Unfallzeitpunkt aktiviert. Das sind im Vergleich zum Gesamtkollektiv 3 % mehr (*p* = 0,596). Eine ICE-Nummer hatten 37 % (*n* = 11) hinterlegt; auch hier zeigt sich eine Steigerung um 10 % (*p* = 0,091). Die ICE war aber bei allen, die keine Notfall-App nutzten, durch eine PIN gesperrt und daher nicht zugänglich.

Bei den Notärzten nahmen insgesamt 103 Personen teil, davon waren 50 % (*n* = 51) Assistenzärzte, 32 % (*n* = 33) Fachärzte und 19 % (*n* = 19) Oberärzte. Von den Befragten gaben 43 % (*n* = 44) an, dass ihnen diese App bekannt sei; 17 % (*n* = 17) würden regelmäßig oder gelegentlich im Notfall auf dem Smartphone nach Informationen suchen. Bereits erfolgreich genutzt wurde die App von 14 % (*n* = 14) der Ärzte. Signifikante Unterschiede zwischen den Erfahrungsstufen zeigten sich hierbei nicht. Von allen Befragten gab nahezu die Hälfte an (*n* = 51; 50 %) regelmäßig und 29 % (*n* = 30) gelegentlich nach Informationen in der Brieftasche und am Körper des Patienten zu suchen. Von den befragten Assistenzärzten gaben 41 % (*n* = 21) an, nach Informationen in der Brieftasche und am Patienten zu suchen, bei den Fachärzten und Oberärzten waren es 57 % (*n* = 30; *p* = 0,528). Die genauen Ergebnisse und Populationsdaten sind in Tab. 3 zusammengefasst.

**Table 3:** 

Teilnehmer	*n* = 192 (*n* = 106 männlich; *n* = 86 weiblich)
Durchschnittsalter	45 Jahre (±15 Jahre)
Smartphonebesitzer	*n* = 174 (*n* = 73 iPhone; *n* = 101 Android)
Notfall-App aktiviert	*n* = 19 (10)
Notfall-App bekannt	*n* = 113 (58)
Notfall-App unbekannt	*n* = 79 (42)
Liste mit Vorerkrankungen	*n* = 41 (21) Durchschnittsalter 56 Jahre (±11 Jahre)
ICE-Nummer gespeichert	*n* = 52 (27)
PIN gesamt	*n* = 161 (84)
PIN und ICE gespeichert	*n* = 45 (86 % von ICE)

Prozente sind in Klammern angegeben

## Diskussion

Die Ergebnisse der vorliegenden Studie belegen, dass bisher weder in der Bevölkerung noch beim medizinischen Personal eine flächendeckende Nutzung der Notfall-App erfolgt ist. So hatten nur 10 % der befragten Patienten in ihrem Smartphone einen aktiven Notfallpass oder die entsprechende App mit Daten bespielt. Hingegen gab die überwältigende Mehrheit der Befragten an, die Notfall-Apps überhaupt nicht zu kennen (41 %), oder diese zu kennen, aber nicht zu nutzen (49 %). Dies könnte insbesondere mit einem fehlenden Risikobewusstsein für ein Unfallgeschehen erklärt werden. Dagegen spricht allerdings, dass knapp ein Drittel aller Befragten eine ICE-Nummer in ihrem Smartphone gespeichert hatte. Dies zeigt, dass durchaus ein Bewusstsein dafür besteht, dass eine Notlage entstehen und das Smartphone in dieser Situation eine Hilfe darstellen kann. Allerdings ist hierbei wiederum von der überwiegenden Mehrheit dieser Befragten (86 %) das Smartphone durch eine PIN geschützt, sodass im Notfall die hinterlegte Nummer nicht ohne Hilfe des Patienten eingesehen werden kann. Es besteht also ein Konflikt zwischen dem grundsätzlich vorhandenen Willen, den etwaigen Helfern Kontaktdaten zur Verfügung zu stellen, und dem Schutz der persönlichen Daten durch eine PIN.

Auch bei jüngeren Patienten (<55 Jahre), die mutmaßlich ein größeres Interesse an Technik haben [8, 9] und sich zunehmend im Internet über ihre Gesundheitsanliegen informieren [10], sind die Notfall-Apps wider Erwarten ebenfalls nur sehr gering (ca. 8 %) verbreitet. Dies könnte unter anderem daran liegen, dass junge Patienten im Alltag tendenziell weniger mit körperlichen Einschränkungen oder gar Leiden konfrontiert werden [5] und sich somit der direkte Nutzen der Notfall-App für sie nicht erschließt. So gaben nach einem Polytrauma immerhin 36 % der Befragten an, dass sich ihre Meinung zu der Verwendung von Notfall-Apps geändert habe. Dies zeigt, dass das unmittelbare Erleben eines schweren Unfalls das Bewusstsein für die Relevanz solcher Apps verändern kann. Dennoch führte ein Unfallereignis nur bei knapp 7 % dieser Patienten zu einer Aktivierung einer Notfall-App. Stattdessen wurde als Reaktion eine Notfallnummer in das Smartphone eingespeichert, die aber – wie bereits in der Einleitung erörtert – in der Regel durch eine PIN gesichert und damit im Notfall nicht abrufbar ist. Insgesamt ist also davon auszugehen, dass bei einem erneuten Unfall – trotz des geschärften Bewusstseins – keine relevanten Informationen abrufbar wären.

Eine alternative Idee, nämlich krankheitsbezogene Daten auf der Krankenkassenkarte zu speichern, lehnten ebenfalls etwa die Hälfte (43 %) der Befragten ab. Ob es sich hier um eine grundsätzliche Skepsis gegenüber der Verlässlichkeit von Krankenkassen und anderen Beteiligten des Gesundheitssystems handelt oder um die allgemeine Angst vor Datenmissbrauch, bleibt unklar und sollte weiter untersucht werden. Zurzeit werden auf den Krankenkassenkarten lediglich folgende Informationen gespeichert: Vor- und Nachname, Geschlecht, Geburtsdatum, Adresse, Versicherungsnummer, Beginn des Versicherungsschutzes, Krankenkasse und die Nummer der Krankenkasse. Ab dem 01.01.2021 sollen diese Daten um bereits vorhandene Notfalldaten und den Bundesmedikationsplan ergänzt werden können. Dies soll nur auf freiwilliger Basis und nach Einwilligung des Patienten geschehen. Wörtlich heißt es im Internetauftritt des Bundesministeriums für Gesundheit: „Patienten können ihren Behandler damit über diese wichtigen Gesundheitsdaten informieren“ [11]. Nach dieser Änderung soll im nächsten Schritt die elektronische Patientenakte vollständig auf der Krankenkassenkarte einsehbar sein. Neben der Patientenakte können dann elektronische Rezepte, eine eventuelle Organspendeerklärung, der elektronische Arzt- und Entlassbrief, die Vorsorgevollmacht und eine Patientenverfügung gespeichert werden. Geplant ist auch, dass der Patient eigenständig Daten, wie etwa Blutzuckertagesprofile, hochladen und verwalten kann. Die Patientenakte soll durch eine PIN geschützt sein, die der Patient zum Auslesen eingeben muss. Die Notfalldaten wie auch der Medikationsplan bleiben für das Rettungspersonal ohne PIN zugänglich. Wie viele Patienten freiwillig ihre Daten hinterlegen lassen, bleibt abzuwarten.

Bei den Notärzten suchen 86 % bei nicht ansprechbaren Patienten nach Informationen, aber nur 5 % davon suchen diese Informationen im Smartphone. Das liegt zum Großteil daran, dass immerhin fast die Hälfte (43 %) der befragten Notärzte die Notfall-Apps überhaupt nicht kennt. Eine Annahme ist, dass einige Ärzte während des Notfalls vergeblich nach Informationen im Smartphone gesucht haben und somit zu dem Entschluss gekommen sind, dass die Suche im Smartphone nur selten zu einem Informationszugewinn führt.

Diese Studie zeigt also, dass auf Seiten der Ärzte bei nicht ansprechbaren Unfallopfern durchaus Informationsbedarf besteht und aktiv danach gesucht wird. Dieser Bedarf wird jedoch nur selten durch tatsächlich hinterlegte Informationen in den Smartphones gedeckt. Die Suche nach einem Medikamentenzettel scheint für die Ärzte lohnenswerter: So sucht etwa die Hälfte der Ärzte regelmäßig nach auf Papier festgehaltenen Informationen. Dies deckt sich mit der bereits beschriebenen Tatsache, dass deutlich mehr Patienten einen Zettel mit Vorerkrankungen bzw. eine Medikamentenliste in der Brieftasche haben als eine aktivierte Notfall-App im Smartphone. Insbesondere bei den über 55-Jährigen lohnt sich der Blick in die Brieftasche, da hier die deutliche Mehrheit (70 %) einen Zettel mit relevanten Informationen bei sich tragen.

Ein Unterschied zwischen dem Verhalten vermeintlicher Notarztanfänger (Assistenzärzte) und den Routiniers (Fachärzte und Oberärzte) konnte nicht festgestellt werden. Die Datenerhebung sowohl zum Kenntnisstand als auch zum Vorgehen bei der Informationsgewinnung war in allen 3 Gruppen gleich verteilt.

Die mediale Aufmerksamkeit bezüglich der Notfall-Apps war bisher eher gering. Zukünftige Aufklärungskampagnen könnten initiiert werden, um die Bekanntheit dieser Apps sowohl in der Bevölkerung als auch beim medizinischen Fachpersonal zu steigern. Dies könnte über die Fachgesellschaften geschehen, die beispielsweise auf ihren Webseiten oder mittels Werbeformaten, wie Informationsbroschüren bzw. Aushängen in den Wartezimmern, darüber aufklären könnten. Immerhin gaben 60 % der Befragten an, dass sie die App aktivieren werden, nachdem sie davon gehört haben. Frühere Kampagnen zur Promotion der Notfallarmbänder zeigten jedoch, dass eine Etablierung mittels Aufklärung und Werbung allein durch die Fachgesellschaften meist keinen signifikanten Anstieg im Bekanntheitsgrad nach sich zog [2]. Innerklinische Aufklärung scheint hier schon das effektivere Mittel, auch wenn eine Reduktion von Mortalität und Morbidität durch die Nutzung entsprechender Armbänder bisher nicht nachgewiesen werden konnte [12]. Auch die Hausärzte könnten bei der Verbreitung eine Rolle spielen. Sie könnten z. B. dazu angehalten werden, die Aufklärung über die Apps in die von den Krankenkassen vorgesehenen Gesundheitschecks zu integrieren.

Unserer Meinung nach ist die Notfall-App eine sinnvolle Ergänzung zu den bereits bestehenden Systemen wie beispielsweise Notizzetteln oder Armbändern. Sollte sich die App zukünftig durchsetzen und an Bekanntheit gewinnen, könnte ein Arzt ohne nennenswerten Zeitverlust Informationen über den zu behandelnden Patienten erhalten und in Notfallsituationen unter Umständen schneller nach Notwendigkeit handeln (z. B. bei Hypoglykämie, Addison-Krise, fehlender Tachykardie bei β‑Blocker-Einnahme).

Dem Nutzer sollte aber bewusst sein, dass die hinterlegten Informationen nicht durch einen Arzt validiert sind. Die Notfall-Apps sind daher konzeptionell und strukturell kein verlässliches medizinisches Tool, sondern eher als Freizeit-App zu werten. Dies kann immer dann zu Problemen führen, wenn die hinterlegten Informationen falsch, ungenau oder lückenhaft sind. Zum Beispiel könnte in der App etwa „Allergie gegen Arzneimittel“ hinterlegt sein. Diese lückenhaften oder falschen Angaben könnten im schlimmsten Fall beim Helfer zur Verunsicherung führen und dem Patienten könnte in Folge dessen eine Therapie vorenthalten werden. Aktuell und unter den bestehenden Voraussetzungen bezüglich der Apps ist es daher nicht möglich, diese Unsicherheiten zu vermeiden. Ein möglicher Lösungsansatz wäre es, dass Hausärzte die Notfall-Apps mit den Patienten gemeinsam ausfüllen und dies in den Apps vermerken, um somit die Informationen zu validieren.

Eine weitere Problematik, die während der Studienrecherche festgestellt wurde, ist, dass der Zugang zu den Notfall-Apps weder standardisiert noch bei jedem Hersteller selbsterklärend, also benutzerfreundlich, ist. Ein solcher – eventuell sogar vorgeschriebener – Standard auf Basis dessen alle Hersteller denselben Zugangsweg zu den Daten etablieren müssten, würde dieser Problematik entgegenwirken. Die Benutzerfreundlichkeit der angebotenen Notfall-Apps wurde in dieser Studie nicht analysiert. Es ist zu empfehlen, dies in weiteren Studien zu untersuchen.

Wir konnten im Rahmen dieser Studie zeigen, dass die Nutzung der Notfall-Apps bei Patienten und Notärzten derzeit unzureichend verbreitet ist. Die mittlerweile standardisierte Sicherung des eigenen Smartphones mittels einer PIN führt zu zusätzlichen Problemen beim Zugriff auf Notfallnummern. Eine breit angelegte Informationskampagne könnte das Bewusstsein für die Notwendigkeit dieser Apps wecken und dabei helfen, durch den erhöhten Bekanntheitsgrad auch die Nutzung der Apps zu steigern.

## Limitationen

In dieser Studie konnten wir nicht überprüfen, ob die in den Notfall-Apps hinterlegten Daten mit den tatsächlichen Patientenbedürfnissen übereinstimmen. Auch konnte aufgrund des Studiendesigns nicht verifiziert werden, ob die Nutzung dieser Apps durch den Notarzt einen tatsächlichen Vorteil für die Behandlung mit sich bringt. Sollten die Notfall-Apps in der Zukunft häufiger genutzt werden, sollte dies in weiteren Studien überprüft werden.

## Schlussfolgerung

Die Notfall-Apps hat aus Sicht der Autoren gefestigtes Potenzial, um zukünftig eine sinnvolle Informationsquelle bei der Notfallversorgung von Unfallopfern zu werden. Aufgrund der geringen Verbreitung und inadäquaten Nutzung der Apps kann Ärzten jedoch zurzeit in zeitkritischen Situationen nicht empfohlen werden, gezielt nach diesen zu suchen.

## Abkürzungen

App: Applikationssoftware (Anwendungssoftware)
ICE: „In case of emergency“
ICE-Nummer: Notfallkontakt (übersetzt)
ISS: Injury Severity Score
PIN: Persönliche Identifikationsnummer
